# Supplementary material for: Antifungal Activities of Essential Oils in Vapor Phase against Botrytis cinerea and Their Potential to Control Postharvest Strawberry Gray Mold
Source: Foods. 2022 Sep 21;11(19):2945. doi: 10.3390/foods11192945 (PMC9563059; doi:10.3390/foods11192945)
Supplement: Supplementary file 1 [file foods-11-02945-s001.zip › foods-1897927-supplementary.pdf]

Table S1. Relative inhibition of *Botrytis cinerea* growth on PDA medium (22±1 °C) after treatment by essential oils (625 ul.L^{-1})

Strain	Kmi-284				Kmi-507				KMi508			
Day	2nd	3rd	4th	7th	2nd	3rd	4th	7th	2nd	3rd	4th	7th
Cardamom	100b	100d	100c	88.3b	100c	100d	100d	100c	100c	100d	100d	100b
Eucalyptus	100b	100d	100c	100c	100c	100d	87c	35.4b	100c	100d	100d	100b
Ginger	100b	81.5c	45.2b	0a	47.5b	30.8b	0a	0a	73.9b	42.5b	30.2b	0a
Grapefruit	100b	75.9b	45.4b	0a	100c	47.2c	43.3b	0a	100c	59.8c	41.3c	0a
Jasmine	-19.4a	-0.6a	0a	0a	7.8a	11.4a	0a	0a	0a	14a	0a	0a
Lemongrass	100b	100d	100c	100c	100c	100d	100d	100c	100c	100d	100d	100b
Levander	100b	100d	100c	100c	100c	100d	100d	100c	100c	100d	100d	100b
Litsea	100b	100d	100c	100c	100c	100d	100d	100c	100c	100d	100d	100b
Mint	100b	100d	100c	100c	100c	100d	100d	100c	100c	100d	100d	100b
Peppermint	100b	100d	100c	100c	100c	100d	100d	100c	100c	100d	100d	100b
Petitgrain	100b	100d	100c	100c	100c	100d	100d	100c	100c	100d	100d	100b
Sage	100b	100d	100c	100c	100c	100d	100d	100c	100c	100d	100d	100b
Thyme	100b	100d	100c	100c	100c	100d	100d	100c	100c	100d	100d	100b

* Values followed by the same letter (in single column) are not significantly different on α=0.05 ANOVA, Tukey HSD post-hoc test.

Table S2. Sensory evaluation of strawberries treated with essential oils after 3 days of storage

	Taste			Appearance			Aroma			Aftertaste			Overall acceptability		
Essential oils	Mean	± SE	Hom. Group	Mean	± SE	Hom. Group	Mean	± SE	Hom. Group	Mean	± SE	Hom. Group	Mean	± SE	Hom. Group
Control	8.47	0.22	c	8.27	0.18	a	8.67	0.16	b	8.67	0.16	c	8.73	0.12	c
Lemongrass	6.66	0.29	b	7.73	0.27	a	7.40	0.38	b	6.47	0.35	ab	7.07	0.32	bc
Litsea	1.6	0.47	b	7.73	0.30	a	7.47	0.38	b	6.87	0.53	b	6.67	0.68	ab
Peppermint	5.53	0.29	ab	7.93	0.23	a	5.73	0.51	a	5.93	0.25	ab	5.00	0.46	a
Thyme	4.87	0.52	a	8.13	0.24	a	5.73	0.52	a	5.20	0.42	a	5.40	0.62	ab

* Values followed by the same letter (in single column) are not significantly different on α=0.05 ANOVA, Tukey HSD post-hoc test.
